# Fabry disease in children: a federal screening programme in Russia

**DOI:** 10.1007/s00431-017-2992-y

**Published:** 2017-09-04

**Authors:** Leyla Seymurovna Namazova-Baranova, Alexander Alexandrovich Baranov, Aleksander Alekseevich Pushkov, Kirill Victorovich Savostyanov

**Affiliations:** 1 Institute of Paediatrics, Federal State Autonomous Institution “National Medical Research Center of Children’s Health” of the Ministry of Health of the Russian Federation, Lomonosovsky prospekt, 2, b.1, 119991 Moscow, Russia; 2 Laboratory of Molecular Genetics and Cell Biology, Federal State Autonomous Institution “Scientific Center of Children’s Health” of the Ministry of Health of the Russian Federation, Moscow, Russia

**Keywords:** Acroparesthesia, Alpha-galactosidase A, Children, Fabry disease, Limb pain, Screening

## Abstract

Our objective was to examine the prevalence of Fabry disease in Russian children with chronic pain in the distal limbs. This non-interventional, multi-centre study included children 2–18 years of age with chronic recurrent unilateral or bilateral pain, burning, or acroparesthesia in the hands or feet. The presence of Fabry disease was defined by abnormal alpha-galactosidase A activity in males or alpha-galactosidase gene (*GLA*) mutation in females. Among 214 patients (110 males), 84.1% had bilateral limb pain and 31.8% had unilateral limb pain recorded at some time point; 61 (28.5%) patients had a positive family history possibly associated with Fabry disease. Alpha-galactosidase A activity was within the normal range in all 109 of the male patients tested. One female patient had a *GLA* mutation (C937G > T) and alpha-galactosidase A activity within the normal range.

*Conclusion*: We did not find definitive evidence of Fabry disease in these children with a history of chronic recurrent unilateral or bilateral limb pain or acroparesthesia. The presence of chronic limb pain does not appear to be highly predictive of a diagnosis of Fabry disease in Russian children and adolescents, suggesting that key early signs and symptoms of Fabry disease are not specific to the disease.

**Table:** 

**What is Known:** • *Signs and symptoms of Fabry disease are seen in children < 10 years of age; pain in the distal limbs is a common early symptom.*
**What is New:** • *Fabry disease was not diagnosed in this population of Russian children with a history of chronic limb pain.* • *The presence of acroparesthesia or chronic limb pain does not appear to be highly predictive of a diagnosis of Fabry disease in Russian children and adolescents, suggesting that these early symptoms of Fabry disease are not specific to the disease.*

## Introduction

Fabry disease (OMIM 301500) is an X-linked lysosomal storage disease associated with a functional deficiency of the lysosomal enzyme alpha-galactosidase A that results in substantial morbidity and premature mortality [16, 17, 26, 28]. Signs and symptoms of Fabry disease can occur in children of both sexes under 10 years of age [26–28].

Classic Fabry disease is seen in hemizygous males with minimal functional alpha-galactosidase A activity. Affected males display the classic phenotype, with the development of characteristic signs and symptoms, including angiokeratomas, acroparesthesia, and hypohydrosis, beginning in childhood and followed by progressive renal, cardiac, and cerebrovascular disease as globotriaosylceramide accumulates in lysosomes throughout the body [4]. Impaired inflammatory and immune responses have been implicated as contributing to disease progression [2, 38].

Other forms of Fabry disease include asymptomatic to severe disease in heterozygous females and later-onset phenotypes associated with renal, cardiac, and cerebrovascular complications in males with various non-classic mutations [4].

Previous reports have estimated the overall frequency of Fabry disease to range between 1 per 117,000 total births [20] and ~ 1 in 40,000 males [4]. Early diagnosis of Fabry disease is often difficult, especially in females, because the symptoms are variable and non-specific [10, 23, 35]. Pain in the distal extremities is a common symptom of Fabry disease, starting in early childhood [37]. Screening for chronic pain in the paediatric population has not previously been explored as a means of identifying patients with Fabry disease.

The primary objective of this study was to assess the prevalence of Fabry disease in an enriched population of paediatric patients with chronic limb pain. A secondary objective was to determine the success rate of study definitions in diagnosing Fabry disease in this population.

## Materials and methods

### Study design and patient population

This was a non-interventional, multi-centre study conducted from December 2013 to March 2015 in seven regions of the Russian Federation with a total population of approximately 38 million: Moscow City, Moscow Region, Saint Petersburg City, Saratov Region, Samara Region, Rostov Region, and Sverdlovsk Regions [30].

Eligible patients were boys and girls 2–18 years of age who presented to a paediatric specialty clinic with a history of chronic (≥ 6 months) recurrent unilateral or bilateral pain, burning, or acroparesthesia in the hands or feet and were considered by the screening physician to be suitable for laboratory testing based on the presence or medical history of additional Fabry disease indicators. These indicators were categorised into three groups and blood sampling was indicated for patients with a single group 1 or group 2 criterion or two group 3 criteria (Table 1). A positive family history was defined as either a family history of Fabry disease or a first-degree relative with a history of renal failure, stroke, or enlarged heart of unknown aetiology.

**Table 1:** Fabry disease indicators for laboratory testing

One criterion from group 1 or 2:		Or two criteria from:
Group 1	Group 2	Group 3
• Positive family history of Fabry disease	• Skin angiokeratomas	• Heat/cold intolerance: positive answer to “Does cold and/or hot weather cause an increase in limb pain or a decrease in the ability to engage in outdoor activities?”
• First-degree relative with history of renal failure, stroke or enlarged heart of unknown aetiology	• Gastrointestinal symptoms: ○ Recurrent abdominal pain: unexplained abdominal pain of ≥ 6-month duration with an average monthly occurrence of ≥ 3 episodes; or ○ Increased stool frequency: bowel movement ≥ 3 times a day (weekly average); or ○ Liquid stool: liquid or watery stool ≥ 2 times a day (weekly average); or ○ Constipation: < 3 bowel movements per week	• Exercise intolerance: positive answer to “Is your child limited in the amount of physical activity he/she can do compared to other children?”
		• Decreased sweating: negative answer to one of the following ○ “When it is hot outside, does your child sweat while doing activities?” or ○ “If your child does sweat, do you think he/she is sweating enough?”

The study endpoint was the presence or absence of Fabry disease, which was defined as abnormal alpha-galactosidase A activity in males or an alpha-galactosidase gene (*GLA*) mutation in females.

### Laboratory testing

Blood samples were drawn to analyse alpha-galactosidase A activity and *GLA* mutations. Alpha-galactosidase A activity was measured in dried blood spots using ultra-performance liquid chromatography–tandem mass spectrometry. Reference values for alpha-galactosidase in our laboratory were determined experimentally and validated according to established protocols.

### Statistical analysis

Descriptive statistics were calculated to evaluate the overall prevalent patient population. A two-tailed Student’s *t* test was used to compare males with females and to compare children (< 14 years of age) with adolescents (≥ 14 years of age).

The prevalence of Fabry disease was estimated by calculating the *FD-R* coefficient:

$$ FD-R=\left[ FD\right]/\left[D\right] $$

where [*FD*] is the number of patients with diagnosed Fabry disease and [*D*] is the number of patients who met the inclusion criteria and were selected for analysis of alpha-galactosidase A activity and/or *GLA* mutations.

Furthermore, the efficacy of the screening method proposed for detecting patients with Fabry disease was estimated by calculating the *Ef* coefficient:

$$ Ef=\left[ FD\right]/\left[C\right] $$

where [*FD*] is the number of patients with diagnosed Fabry disease and [*C*] is the number of patients 2–18 years of age who reported to a specialty clinic with pain as the chief complaint and with chronic (≥ 6 months) unilateral or bilateral pain, burning, or acroparesthesia of unknown cause in the hands or feet.

## Results

### Patients and demographic characteristics

A total of 214 patients (110 males and 104 females) fulfilled the inclusion criteria and were selected for screening. Of these, 84.1% had bilateral limb pain recorded at some point and 31.8% had unilateral limb pain recorded at some point. No statistically significant differences were observed in age or ethnicity between males and females (Table 2).

**Table 2:** Demographic and clinical characteristics

Characteristic	Female *n* = 104	Male *n* = 110	Overall *N* = 214
Age, median (range) years	10.4 (2.1–17.9)	10.6 (2.2–17.9)	10.6 (2.1–17.9)
Age group < 14 years, *n* (%)	79 (47.6)	87 (52.4)	166 (100)
Age group ≥ 14 years, *n* (%)	19 (39.6)	29 (60.4)	48 (100)
Caucasian, *n* (%)	102 (98.1)	106 (96.4)	208 (97.2)
Recurrent unilateral limb pain/acroparesthesia, *n* (%)^a^	38 (46.3) *n* = 82	30 (40.0) *n* = 75	68 (43.3) *n* = 157
Recurrent bilateral limb pain/acroparesthesia, *n* (%)^a^	88 (85.4) *n* = 103	92 (86.0) *n* = 107	180 (85.7) *n* = 210
General gastrointestinal disorders or recurrent abdominal pain, *n* (%)	78 (75.0)	73 (66.4)	151 (70.6)
Recurrent abdominal pain of unknown origin, *n* (%)	48 (46.2)	55 (50.0)	103 (48.1)
Angiokeratomas, *n* (%)	11 (10.6)	10 (9.1)	21 (9.8)
Heat and/or cold intolerance, *n* (%)	40 (38.5)	57 (51.8)	97 (45.3)
Exercise intolerance, *n* (%)	72 (69.2)	73 (66.4)	145 (67.8)
Anhydrosis or hypohydrosis	29 (27.9)	40 (36.4)	69 (32.2)

^a^Percentages based on number of patients with available data

### Signs and symptoms of Fabry disease

**Fig. 1:**
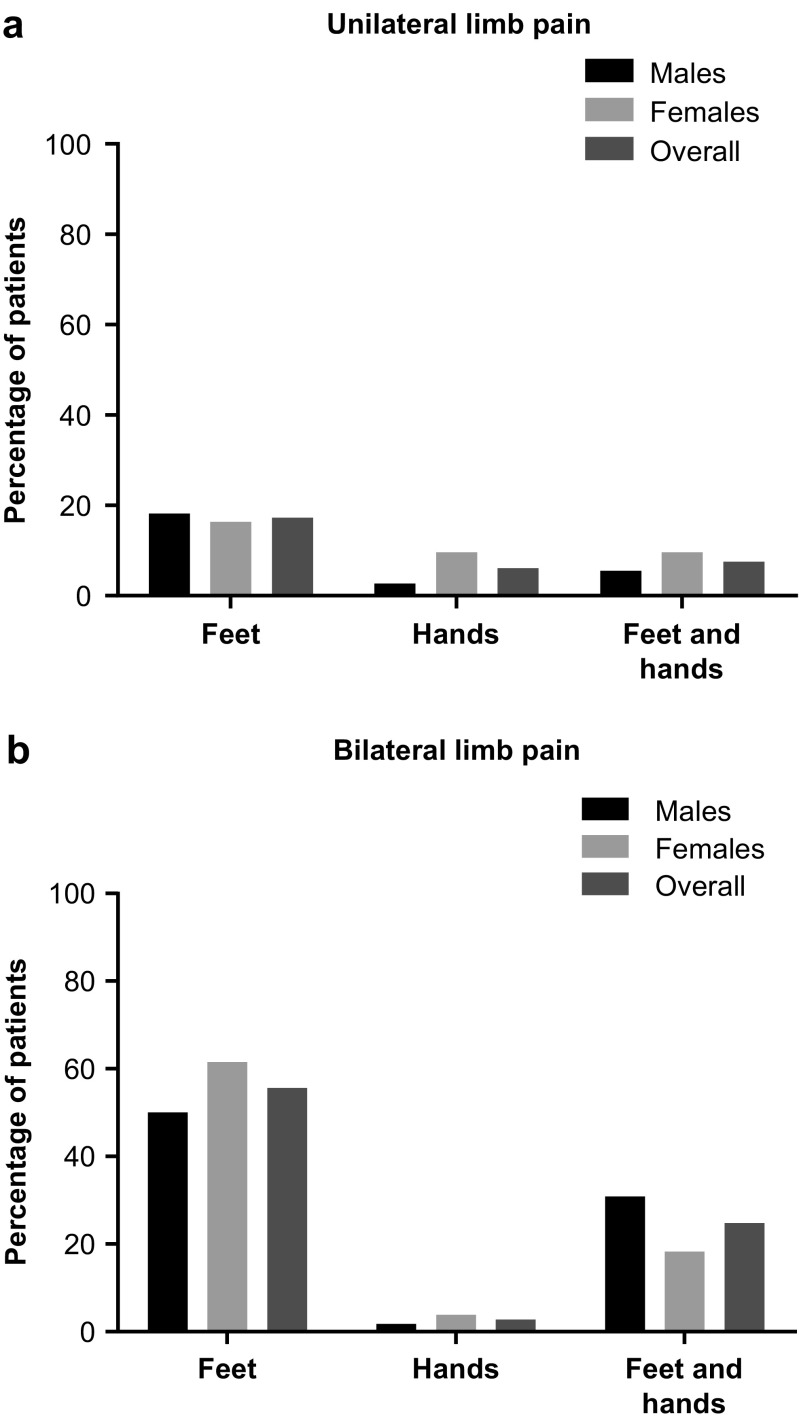
Recurrent limb pain/acroparesthesia by location. **a** Unilateral. **b** Bilateral

**Fig. 2:**
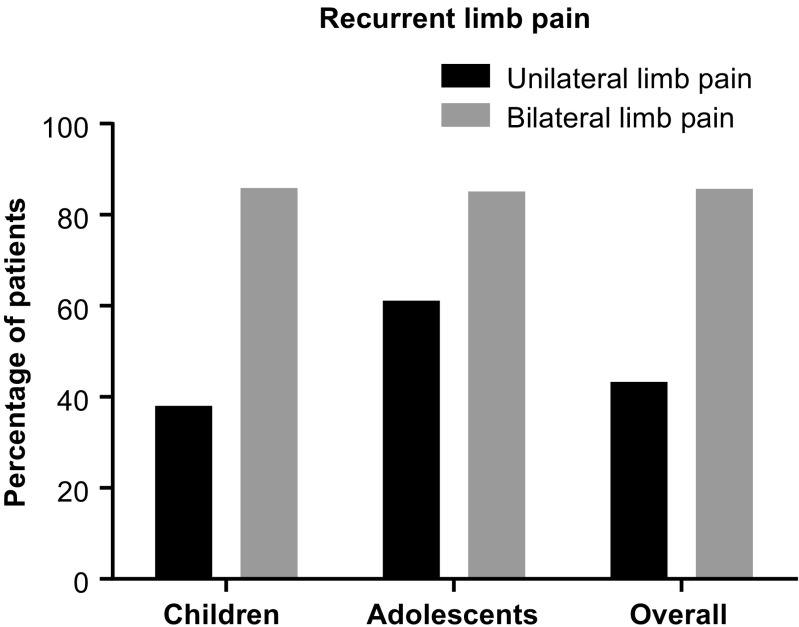
Recurrent unilateral and bilateral limb pain/acroparesthesia by age group

The prevalence in females and males of chronic recurrent unilateral and bilateral limb pain/acroparesthesia is shown in Table 2; prevalence by location is shown in Fig. 1. Recurrent unilateral limb pain/acroparesthesia was reported less frequently in children (38.0%) than in adolescents (61.1%; *p* = 0.014; Fig. 2).

The mean (standard deviation [SD]) intensity of limb pain on a 10-point scale was 5.1 (1.7) in the overall population. Mean [SD] intensity of limb pain was significantly higher in females (5.3 [1.7]) than in males (5.0 [1.6]; *p* = 0.024) and was significantly higher in adolescents (5.5 [1.4]) than in children (5.0 [1.7]; *p* = 0.041).

As a whole, general gastrointestinal disorders or recurrent abdominal pain were observed in 75.0% of girls and 66.4% of boys (*p* = 0.17; Table 2). Recurrent abdominal pain of unknown origin was seen in approximately 48% of the patients overall (Table 2), and the prevalence was not significantly different between females and males (*p* = 0.40). The prevalence of various signs and symptoms commonly associated with Fabry disease is shown in Table 2.

### Family history

Overall, 61 (28.5%) patients had a positive family history possibly associated with Fabry disease. None of the patients had an immediate relative with a diagnosis of Fabry disease. There were six (2.8%) patients (three females, three males) who had relatives with current renal failure, and one (0.5%) patient had a relative who died from renal failure. A total of 45 (21.0%) patients (27 females, 18 males) had relatives with premature cardiovascular disease (< 50 years of age), and 22 (10.3%) had a relative who died from cardiovascular disease younger than 50 years of age.

### Laboratory testing

Alpha-galactosidase A activity (Table 3) was within the normal range in all 109 of the male patients tested. A mutation of *GLA* (C937G > T) was detected in only one of the 104 female patients tested. This patient had bilateral daily pain in her feet for > 10 years, exercise intolerance, heat/cold intolerance, and anhydrosis. Her alpha-galactosidase A activity was considered to be within the normal range (the specific value was not available).

**Table 3:** Blood testing for alpha-galactosidase A activity in males

Alpha-galactosidase A, μmol/L/h	*n* = 109
Minimum	2.38
Maximum	25.08
Median	4.62
Mean	5.93
Standard deviation	3.80

### Integral study results

The coefficient *FD-R*, to characterise the prevalence of Fabry disease, was calculated as 1/261 ≈ 0.0038. This is equivalent to 38 patients with Fabry disease per 10,000 patients in the enriched population of paediatric patients with chronic limb pain.

The coefficient *Ef*, to characterise the efficacy of the screening method used for the detection of patients with Fabry disease, was calculated as 1/1736 ≈ 0.00058. This is equivalent to 58 patients with Fabry disease per 100,000 patients with chronic pain, burning, or acroparesthesia of unknown aetiology in the hands or feet.

## Discussion

We did not find definitive evidence of Fabry disease in this enriched population of Russian children with a history of chronic recurrent unilateral or bilateral limb pain or acroparesthesia. Gastrointestinal disorders, angiokeratomas, heat and/or cold intolerance, and anhydrosis or hypohydrosis, all commonly reported features of classic Fabry disease, occurred at frequencies varying from 9.8 to 75% overall. This may indicate that such features are not specific to Fabry disease when aligned with chronic recurrent limb pain or acroparesthesia. Gastrointestinal disorders, in particular, are non-specific symptoms that are particularly prevalent in the general paediatric population. This is reflected in our study, with gastrointestinal symptoms reported in ~ 70% of children.

The non-pathogenic *GLA* mutation C937G > T was detected in only one symptomatic girl with normal alpha-galactosidase A activity. This is classified as a “pseudodeficiency” mutation and has been described in individuals with normal or somewhat decreased alpha-galactosidase A activity without other evidence of Fabry disease [7, 8]. Diagnosis of Fabry disease is especially difficult in females, as enzyme levels alone are generally not sufficient and genetic analysis is normally required [10, 35].

Screening studies for Fabry disease have previously been reported in other subpopulations. For example, in a study of patients with small-fibre neuropathy, 5/24 (20.8%) were found to have *GLA* mutations [34]. In patients with left ventricular hypertrophy, the prevalence of Fabry disease has been variously reported between 0.5 and 11.8% [1, 6, 15]. In young adult patients with stroke, Fabry disease prevalence has been reported to be between 0.3 and 2.8% overall [9, 29, 36], and to be 4.2% in men and 2.1% in women [15]. Atypical variants of Fabry disease associated with renal involvement may be diagnosed, and functional variants of *GLA* may be present in patients with end-stage renal disease [5, 13, 14, 21]. Furthermore, the prevalence of Fabry disease among dialysis patients has been variously reported as 0.33 to 3.2% in men, 0.10% in women [15, 18], and 0.02 to 0.32% overall [21, 22, 31].

The incidence of Fabry disease in the general population has been estimated as about 1 in 40,000 males [4]. Studies from different countries report varying frequencies of Fabry disease. Based on data from the Netherlands during the period 1970 to 1996 (covering birth years 1926 to 1986), the prevalence was estimated to be 0.42 per 100,000 (or ~ 1 per 238,100) male births [25]. Fabry disease prevalence was estimated to be ~ 1 per 117,000 births during the period 1980 through 1996 [20] in Australia and to be 0.12 per 100,000 (or ~ 1 per 833,000) live births in northern Portugal [24]. In a UK study, the prevalence among male individuals was estimated to be ~ 1 in 366,000 [16].

Screening of 37,104 consecutive newborn males in Italy found that ~ 1 in 3100 males had Fabry disease based on abnormally low alpha-galactosidase A activity levels and the presence of alpha-galactosidase A mutations [33]. The same study estimated the prevalence of classic Fabry disease as ~ 1 in 37,000 newborn males in Italy [33]. In Taiwan, enzyme-based screening of 171,977 infants at 3 days of age indicated an overall frequency of ~ 1 in 1250 males and ~ 1 in 40,840 females [11], and classic Fabry disease was estimated to occur in about 1 in 22,570 newborn males [11]. In other studies, the screening of 21,170 newborn infants in Japan showed a prevalence of 1 in 7057 [12]; laboratory screening of ~ 54,800 newborn males in the US state of WA revealed a prevalence of ~ 1 in 7800 [32]; and screening of 34,736 consecutive newborn infants in Austria found mutations characteristic of Fabry disease in 1 per 3859 births [19].

The prevalence of non-classic or later-onset Fabry disease has also been investigated. For example, the prevalence of carriers was estimated to be ~ 1 in 339,000 females in the UK [17]. In a screening study of newborn males in Italy, ~ 1 in 3100 to 4600 was identified with a later-onset phenotype, giving a ratio of approximately 7:1 of patients with a later-onset:classic phenotype [33]. In Taiwan, the estimated frequency of the later-onset phenotype was ~ 1 in 1390 newborn males; for the IVS4 mutation it was ~ 1 in 1460 newborn males on the basis of enzyme-based screening [11]. Subsequent DNA-based screening in 20,063 newborn males and females found the prevalence of the IVS4 mutation to be ~ 1 in 875 males and ~ 1 in 399 females [3].

This is the first analysis of Fabry disease prevalence in an enriched population of paediatric patients with chronic limb pain. Patient numbers were relatively low, although this is to be expected in rare disease studies. Fabry disease was not diagnosed in this population of Russian children with a history of chronic limb pain. The presence of acroparesthesia or chronic limb pain does not appear to be highly predictive of a diagnosis of Fabry disease in Russian children and adolescents, even after applying a Fabry disease-specific enrichment protocol, raising the question of the specificity of these indicators and suggesting that key early signs and symptoms of Fabry disease are not specific to the disease. Future studies, including the detection of polymorphisms and pathogenic variants in *GLA*, will permit analysis of genotype-phenotype correlations and should allow us to apply a personalised approach to the management of these patients in the future; these may also allow the identification of other biomarkers and the creation of a diagnostic algorithm based on a mathematical model of various indicators suggesting a differential diagnosis of Fabry disease.

## Abbreviations

*GLA*: alpha-galactosidase gene
SD: standard deviation
